# Unveiling horizontal gene transfer in the gut microbiome: bioinformatic strategies and challenges in metagenomics analysis

**DOI:** 10.1093/nsr/nwaf128

**Published:** 2025-04-01

**Authors:** Haoran Peng, Jingyuan Fu

**Affiliations:** Department of Genetics, University of Groningen, University Medical Center Groningen, the Netherlands; Department of Genetics, University of Groningen, University Medical Center Groningen, the Netherlands; Department of Pediatrics, University of Groningen, University Medical Center Groningen, the Netherlands

Microorganisms can inhabit diverse environments, including the human gut [1]. Gut bacteria can continuously evolve in response to changes in the intestinal environment, which can be linked to the host's metabolic and immune status, lifestyle and environmental exposures, including drugs and dietary intake. As a result, adaptive genetic variants are widely observed in gut bacterial genomes, including both single nucleotide variants (SNVs) and genomic structural variants (SVs) [2,3]. SNVs likely arise from single nucleotide mutations, with the typical genomic mutation rate of bacteria estimated to be ∼0.001 per generation [3]. SVs are larger genomic segments that can be deleted, reversed or duplicated [2]. Several methods have recently been developed to profile SVs in the gut microbiome, shifting gut microbiome from species to genetic level [2]. Analysis of genetic variation in the gut microbiome may be helpful for discovering intraspecies variation and bacterial functional genes that are related to human host genome, lifestyle and health [2]. In particular, the genetic differences in the large genomic segments in SVs make their biological interpretation easier than that of SNVs. For example, deletion of SVs can result in the absence of specific genes or pathways, effectively functioning as naturally occurring gene knockouts, while variable SVs can be associated with changes in the abundance of genes or pathways, suggesting naturally occurring upregulation or downregulation. We previously employed SV-based approaches to pinpoint the novel bacterial GalNAc utilization pathway associated with host blood type [4].

Different mechanisms have been suggested to underlie bacterial SVs, including homologous and non-homologous recombination, transposition and horizontal gene transfer (HGT) [2]. HGT in particular is considered one of the most important evolutionary mechanisms for bacteria, allowing genetic materials to be transferred across different bacteria [5]. Genetic materials that can move around within a genome or be transferred across different genomes are called mobile genetic elements (MGEs), while genomic islands (GIs) are MGEs larger than 10 kb that are integrated into the bacterial chromosome (Box 1). Through HGT, bacteria can acquire ‘beneficial’ DNA, particularly in their accessory genomes, via transduction, conjugation (Fig. 1A) and outer membrane vesicles. This can change bacterial functionality, enhancing their fitness and adaptation to environmental stresses, and influence microbe–microbe and microbe–host interactions [5]. Ares-Arroyo *et al.* introduced the concept of ‘hitcher’ genetic elements, which constitute a large fraction of the pathogenicity and resistance GIs [6]. Hitcher elements can convert bacteria from being mutualistic to pathogenic by introducing virulence factors [6]. Evidence from *Shewanella xiamenensis* has demonstrated the role of HGT in genetic diversity, pathogenicity and adaptive evolution in bacterial symbionts and host–microbe symbioses [7].

**Figure 1.**
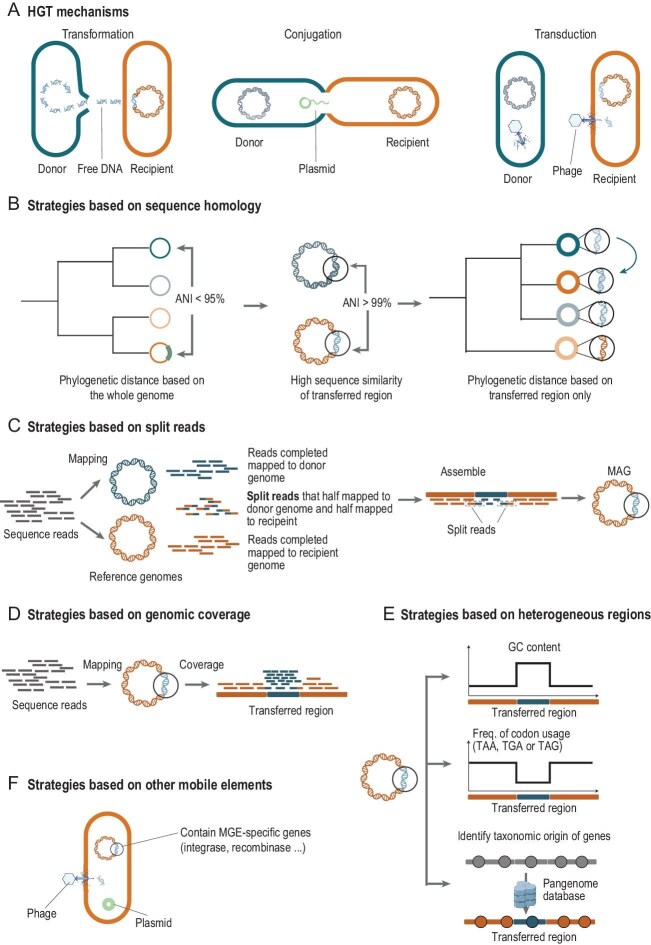
Different strategies for detecting horizontal gene transfer (HGT). (A) HGT mechanisms. The main HGT mechanisms include transformation, conjugation and transduction. Transformation (left) is the process of absorbing free DNA from the environment, which is limited by DNA homology. The higher the homology between free and host DNA, the easier it is to integrate into the host genome. Conjugation (middle) is primarily mediated by plasmid, which facilitates the transfer of DNA through conjugative pili. However, integrative and conjugative elements can also facilitate conjugation and be integrated into bacterial chromosomes. Transduction (right) is a process mediated by bacteriophages, which can integrate into the host genome and carry host DNA for further dissemination. The host DNA carried varies depending on the type of transduction mechanism. Specialized transduction can only carry host genes that neighbor the prophage, whereas generalized transduction can carry genes from throughout the host and lateral transduction can carry large segments of host genes that neighbor the prophage. The donor genome is in blue and the recipient genome is in orange, with the transferred region from the donor highlighted in blue. (B–F) Strategies to identify HGT based on specific features. (B) Strategies based on sequence homology. To infer HGT, this approach identifies nearly identical DNA in distantly related genomes (e.g. based on their phylogenetic distance). For instance, if the phylogenetic distance at the whole-genome level is high (average nucleotide identity (ANI) > 95%), while the phylogenetic distance of the transferred region is low (ANI > 99%), this region is likely to be transmitted. Comparing the species tree and the gene tree can infer the direction of transfer. (C) Strategies based on split reads. In this approach, sequence reads are mapped to reference genomes and a set of reads are defined as ‘split reads’ when they can be divided into two parts that each map to a different genome. After assembling and binning, the regions flanked by these split reads represent the transferred region. (D) Strategies based on genomic coverage. Due to the integration of donor DNA, reads mapped to HGT regions often show distinct sequencing coverage patterns compared to those mapped to other genomic regions. (E) Strategies based on heterogeneous regions. These approaches are based on the fact that the horizontally transferred region and the rest of the recipient genome can have a significantly different sequence nucleotide composition, exemplified by variations in GC content, codon usage and the taxonomic origin of genes. (F) Strategies based on other mobile elements. HGT regions are likely to be mobile genetic elements (MGEs), which can be characterized by the presence of plasmids, phages and specific genes such as integrase and recombinase.

HGT in the human gut microbiome has also been found to be closely related to human lifestyle and health. For example, the gut microbiome of individuals from an agrarian population in Fiji showed an enrichment of genes for plant-based starch degradation [8]. In contrast, gut bacteria in individuals with industrialized lifestyles appear to acquire new functionality through high rates of HGT [9]. Energy sources for gut microbes can also come from the host. For instance, having A blood type and FUT2 secretor status, both determined by host genetics, determines the availability of GalNAc in intestinal mucus, thereby influencing the presence rate of a bacterial genomic element containing the GalNAc utilization pathway. MGEs from the maternal microbiome have also been shown to shape the metabolic potential of the infant gut microbiome via HGT [10]. A better understanding of HGT in the human gut microbiome would thus help unravel a driving force behind the functional variation within and between species, as well as within dynamic host–microbe interactions. This can be achieved by uncovering the biological processes through which functions are acquired and by establishing associations with the host's lifestyle, environmental exposures, physiological parameters and health status.

The classic way to study microbial HGT is to use reporter genes, constructed in plasmids or bacteriophages,

Box 1. **Terminologies related to HGT.**

**Horizontal gene transfer (HGT)** (also known as lateral gene transfer): A process in which an organism transfers genetic material to another organism outside of parent–offspring inheritance (vertical transmission). HGT is an important adaptation mechanism by which bacteria can acquire new functionalities to expand their metabolic capabilities, deal with environmental stresses, enhance virulence, etc. There are three different mechanisms of HGT: transformation, transduction and conjugation.

**Mobile genetic elements (MGEs)** (also known as selfish genetic elements): These are genetic materials that can move within a genome or across different genomes. MGEs include transposons, integrons, phages and plasmids, which can all serve as vehicles for genetic exchange. HGT mechanisms often involve MGEs. For instance, bacteriophages mediate transduction, while plasmids are involved in conjugation.

**Genomic island (GI):** These are a specific subset of MGEs, typically a large genomic segment (>10 kb) that is horizontally transferred and integrated into the bacterial chromosome. GIs often contain genes that are not essential for bacterial survival but confer adaptive advantages under specific conditions. GIs also exhibit special sequence characteristics, including variations in GC content, codon usage and the presence of mobility-relevant genes. Their movement and integration are often mediated by other MGEs such as integrases, transposons or conjugative elements.

for detection through fluorescence or antibiotic selection [5]. Improvements in culturomics now also enable further comprehensive study of HGT. Finally, with the development of sequencing technology, comparative genomics based on whole-genome sequencing of different bacterial species in culture has been an appealing approach for examining HGT [1]. This approach identifies highly identical (>99%) DNA segments shared between distant species, as defined by an average nucleotide identity < 95% or a 16S rRNA gene similarity < 97% between species. Through this approach, we have now learned that HGT is more likely to occur between phylogenetically related, highly abundant bacteria that co-occur in a microbial community [9,11]. Despite these advances, culture-based approaches for studying HGT in the human gut microbiome are currently limited as most gut bacteria remain difficult to culture and *in vitro* conditions cannot fully mimic the *in vivo* intestinal environment or recapitulate the entire microbial community [5]. However, the large amount of shotgun metagenomics sequencing data now being generated for human fecal samples, combined with development of bioinformatic tools to probe this data, should open up new opportunities to assess HGT in human gut bacteria at an unprecedented scale. In this perspective, we focus on the bioinformatic tools developed to detect HGT in metagenomic data (Table S1) and the new insights they bring. We have grouped these tools into different categories based on the main features of HGT they capture, although individual tools can combine multiple strategies.

**Strategies based on sequence homology:** Horizontally transferred genes exhibit high homology across genomes and low homology to flanking DNA, although genes with high homology can also be mistaken for conserved genes [1], raising questions about whether our definition of conserved genes might be conflated with HGT. The simple, conservative comparative genomic strategy when using whole-genome sequences is to identify nearly identical DNA in distantly related organisms [5]. However, this does not capture HGT among closely related species. To solve this, the populations as clusters of gene transfer (PopCOGenT) approach uses an alternative method that infers HGT by detecting regions with low genetic variation (likely acquired through HGT) and regions with high genetic variation (likely inherited from ancestor to descendant) between genomes in phylogenetically close bacteria. PopCOGenT is a powerful tool for detecting HGT in closely related species, but not in the genomes of distant ones. A more flexible tool, Rapid ANalysis of Gene family Evolution using Reconciliation-DTL (RANGER-DTL), infers HGT in both closely and distantly related organisms by comparing the rooted phylogenetic tree constructed from their conserved genes (or whole genomes) with trees built from the putative HGT region. In brief, the phylogenetic distance between the recipient and donor organisms is distinct, placing them in different clades of the phylogenetic tree. However, transferred regions will exhibit high sequence similarity between the two genomes. Consequently, when considering only the phylogenetic distance of the transferred region, the recipient genome would cluster within the same clade as the donor genome (Fig. 1B, Table S1).

While both PopCOGenT and RANGER-DTL perform well on whole-genome sequences, the availability of bacterial isolates from the human gut microbiome remains limited. To enable HGT analysis in metagenomics data, metagenomics Community-level identification of HGTs (MetaCHIP) is the first tool that can detect HGT in assembled genomic contigs or metagenome-assembled genomes (MAGs) from short sequencing reads [12]. It combines the comparative genomic and phylogenetic tree comparison strategies to detect HGT. The advantage of this approach is that the timing of transfer events can be inferred based on the similarity of homologous genes. Using this strategy, Song *et al.* demonstrated that HGTs predominantly carried various mobilomes, including prophages and transposons. Messerer *et al.* found that HGTs from different historical eras are enriched with distinct functions. Recent HGTs (0–10 000 years ago) are enriched for defense mechanisms, intracellular trafficking and secretion, whereas ancient HGTs (>10 000 years ago) are primarily enriched with genes related to metabolism [11]. By constructing a pangenome for each species, the authors also observed that the probability of encountering a transferred gene in the cloud genome (the accessory genome) is more than twice that of encountering a non-transferred gene [13].

**Strategies based on split sites**: Transferred sequences have special split sites, resulting in non-homologous regions flanking highly homologous transferred regions (Fig. 1C). When mapping shotgun metagenomic reads to reference genomes, a sequenced read that can be divided into two parts, each mapping to a different genome, is considered a split read. The region between two split reads then indicates the transferred region (Fig. 1C). The Daisy method detects HGT based on this strategy. Daisy first identifies HGT boundaries with split reads and then filters candidate regions using coverage and read pair information. The distribution of read coverage across the genome can be used to further validate HGT based on the assumption that the transferred region in the donor should have similar coverage to other regions in the recipient. However, Daisy's effectiveness in detecting split reads depends on the availability of accurate genomes for both donor and recipient organisms. LEMON solves this issue by clustering split reads using the DBSCAN (density-based spatial clustering of applications) algorithm to identify candidate HGT breakpoints, so it does not need to know donor and recipient genomes in advance, making it suitable for analyzing metagenomic data (Table S1). More recently, the same research group developed a new tool, LocalHGT [14], which further improves computational efficiency by introducing a k-mer-based fuzzy matching approach for breakpoint discovery, thereby reducing CPU usage by 82%. This can allow fast HGT detection in a large number of metagenomic samples, enabling HGT-based microbiome epidemiology studies. LocalHGT's utility has been shown by HGT detection in >2000 human gut microbiome samples, with results showing that HGT profiles could be host-specific. Differential HGTs can be used for disease prediction, as well as for functional implication in disease etiology.

**Strategies based on genomic coverage:** Due to HGT, a DNA segment may be present in both donor and recipient genomes. Therefore, HGT regions often show distinct sequencing coverage patterns compared to other regions of the donor/recipient genome (Fig. 1D), resulting in detection of structural variation. The tool SGVFinder was designed to identify SVs by aligning metagenomic reads to species-representative genomes and calculating the coverage variation across genomic regions [2]. While SGVFinder was not developed specifically to detect HGT, distinct sequencing coverage regions may indicate multiple copies or deletion of genomic regions, which can be partially explained as outcomes of HGT. For instance, we used SGVFinder to detect the SV region in *Faecalibacterium prausnitzii* that harbors the GalNAc utilization pathway [4]. Interestingly, we also found that this SV region is likely to be an MGE and that SV-sharedness is higher between co-housing individuals [4]. This strategy can therefore be used as a supplementary method to validate gene transfer. WAAFLE (workflow to annotate assemblies and find lateral gene transfer event), Daisy, LEMON and LocalHGT all employ a similar strategy to enhance the reliability of the transferred regions they identify (Table S1). After identifying HGT regions, these tools further map the distribution of coverage of sequencing reads on the donor and recipient genomes (especially the distribution of HGT regions) to see whether the coverage of the HGT region differs significantly.

**Strategies based on heterogeneous regions:** HGT results in heterogeneous regions in the recipient bacterial chromosome that may exhibit a nucleotide sequence composition significantly different from the rest of the genome. This is exemplified by variations in dinucleotide frequency, guanine-cytosine (GC) content, and codon usage [5] (Fig. 1E). Several tools were developed to compare the sequence characteristics across the genome to detect heterogeneous regions. One particular example used to infer GIs, Islandviewer, is a web-based server that incorporates three types of GI-detection method: IslandPath-DIMOB based on nucleotide composition and the presence of mobility genes, SIGI-HMM based on codon usage bias, and IslandPick based on a comparative genomics approach (Table S1). The ensembled method Islandviewer has been used to perform more comprehensive classification of GIs. Recently, DICEP (detection of islands by composition, enrichment and phylogenetics) was developed as a powerful combined method for GI prediction. DICEP jointly considers specific characteristics of GIs, including compositional bias, unusual phylogenetic patterns and marker gene enrichment. GIs are large HGT regions (normally >10 kb), and the sensitivity of these GI-detection methods can be affected by the size of assembled genomic contigs (Table S1). Alternatively, genomic heterogeneity can also be assessed for individual genes at the level of potential species origin through a homolog-based search. This is the specific strategy of WAAFLE [15]. The WAAFLE developers first constructed pangenomes for over 4000 species; WAAFLE uses this to perform a homolog-based search and assign each gene from a specific genomic contig to its potential taxonomic origin. If a gene's taxonomic origin differs from that of other genes, WAAFLE suggests it as likely to be transferred. Since the constructed pangenome also includes species outside the sample's microbial community, WAAFLE is well-suited to detect HGTs beyond a single community sample. Using this method, Hsu *et al.* profiled HGT events in the human microbiome in >2000 metagenomic samples from various human body sites and found that HGT events were highly specific to each site [15]. They also showed that HGTs are enriched in MGEs and genes with transport functions and that highly abundant species are more likely to act as donors in HGT events.

**Strategies based on other MGEs:** HGT can be mediated by MGEs, particularly through phages and plasmids, so the transferred regions often contain specific genes (Fig. 1F). For example, phage-mediated HGT regions contain phage integrase, while conjugation involves mobilization genes and integrative and conjugative elements. Recombinase and integrase can serve as additional indicators of HGT regions [5]. Many tools, like IslandPath-DIMOB, have incorporated the identification of MGEs for their HGT region detection, while other tools were specifically designed to detect plasmids and phages. For instance, geNomad used a hybrid approach to identify plasmids and phages that were based on genetic markers and alignment-free approaches (Table S1). But it cannot distinguish linear or circular plasmids. In contrast, PlasX is a machine-learning-based tool designed for plasmid detection that utilizes deep-learning techniques to distinguish plasmid sequences from chromosomal DNA [16]. PlasX improves the efficiency of plasmid detection by constructing *de novo* gene families from 16 827 plasmids and 14 367 chromosomal sequences for model training. By doing so, PlasX identified over 68 000 non-redundant plasmids in the human gut microbiome. The advantage of this strategy for HGT detection is that it can directly infer the transfer mechanisms (transduction, conjugation) through bacteriophages or plasmids. For example, a recent study using PlasX found a cryptic plasmid, pBI143, that is widespread in industrialized populations and replicates rapidly in patients with inflammatory bowel disease [17]. Although there is currently no evidence to support pBI143’s role in bacterial host fitness, there are data supporting its potential to track human colonic inflammatory states [17].

**Prospectives in studying HGT in the human gut microbiome:** The human gut microbiome is a dynamic ecosystem. With increasing collection of longitudinal microbiome samples from human cohorts, research has shifted from static description of the gut microbial composition to studying its dynamic interaction and adaptation over time, as well as its relevance for host health [18]. HGT is clearly one of the notable processes that drives microbial interaction and adaptation. We note, however, that the human gut environment is a unique niche where microbial HGT may show different patterns compared to other environments. While our understanding of the frequency and distribution of HGT is improving, several key questions remain unanswered with regard to the human gut microbiome.

*What is the impact of HGT in gut microbial interaction and resilience?* HGT can facilitate the coexistence of different bacteria through gene-sharing, thereby stabilizing microbial communities and enhancing microbial resilience [19]. We can thus hypothesize that a community with a higher frequency of HGT may show a higher level of resilience. However, to what extent HGT contributes to the stability and resilience of the microbial community remains unclear. While genes involved in HGT are often related to antibiotic resistance, virulence, pathogenicity and metabolism, their specific roles and contributions in microbial interaction and resilience have not been thoroughly explored [18].

*What are the driving mechanisms of HGT in the human gut microbiome?* Numerous mechanisms drive HGT, including plasmids, phages, homologous recombination and vesicles, yet it remains uncertain which most effectively facilitates gene transfer within a gut microbial community and across different individuals. It is also unclear whether super-mobile elements that show exceptionally high capacity to move or transfer genes exist in the human gut microbiome. Different bacteria utilize different HGT mechanisms, which can have different effects on their adaptation and colonization in the gut [18]. Further, it is worth noting that bacteria-targeted metagenomic sequencing has limited recovery for plasmids and the gut virome, so their comprehensive profiling requires specialized techniques for DNA/RNA extraction.

*What is the underlying natural selection and evolutionary mechanism of HGT?* The Baas-Becking hypothesis that ‘everything is everywhere, but the environment selects’ suggests that both gene transfer rate and natural selection contribute to the observed HGT frequency [20]. For the human gut microbiome, such environmental selective pressure might be related to aspects of the host's lifestyle. Antibiotic usage, smoking, alcohol consumption, diet and other medication use are already known to significantly impact the gut environment, thereby influencing the adaptation of gut bacteria via HGT and shaping the functionality of the gut microbiome [9]. Moreover, HGT rate can be influenced by other evolutionary mechanisms such as population bottlenecks and drift, host–microbe co-evolution, adaptive radiation, gene flow and human-to-human strain transmission [21]. However, analyses that assess human gut microbial HGT dynamics, and its relevance to human genome, lifestyle and health, are still limited.

*What is the relevance of HGT in human health?* HGT in the human gut microbiome exhibits significant functional differences between ancient and recent gene transfers [11]. Inter-individual differences should be enriched for recent HGTs, which reflect the dynamic gene transfer flow within an individual's gut community. Evidence supports the role of HGTs in colorectal cancer and acute diarrhea, and HGT patterns have shown potential as a biomarker for colorectal cancer prediction [14]. However, studies that link HGT in the human gut microbiome to host health are still scarce.

To answer these questions, it is crucial to comprehensively profile HGT patterns in individual gut microbial communities and to integrate those patterns with deep phenotype data in large-scale human studies. The growing number and completeness of reference genomes available for human gut microbiota are also enhancing our capacity to study HGT. Above we discussed different strategies for HGT detection that can be applied to MAGs constructed in individual microbial samples, and the advantages and limitations of each strategy are briefly summarized in Table S1. Most tools, if applicable to MAGs, are affected by MAG quality. First, due to modest sequence depth per sample, the number of high-quality MAGs obtained from a single sample is still low, which limits the power of MAG-based HGT analysis at the individual level. Second, MAGs are constructed through assembling short sequence reads and binning genomic contigs. On the one hand, sequence heterogeneity due to HGT, such as diverging GC content and varying genomic coverage, can influence MAG construction. As a result, contigs containing heterogeneous regions may not be binned with the rest of the genome [5]. On the other hand, reads from HGT regions can map to multiple genomes, leading to inaccurate binning and the formation of incomplete or incorrect MAGs, and this contamination (heterologous sequences) can be misidentified as HGT [22].

To overcome the technical challenges related to MAGs based on short reads, we need to refine existing methodologies and develop new ones. Some questions will be addressed when more advanced sequencing technologies emerge. For instance, long-read sequencing can provide high-throughput, low-contamination genomic content [23]. Hi-C metagenomic sequencing uses a proximity ligation method to infer the bacterial origins of plasmids, antibiotic resistance genes (ARGs) and HGT regions [24]. Moreover, MGEs and their bacterial hosts can also be simultaneously visualized by combining single-molecule DNA fluorescence *in situ* hybridization (FISH) with multiplexed ribosomal RNA-FISH [25]. A high-throughput single-cell sequencing method, Microbe-seq, has now revealed that 80% of HGT sequences include MGEs [26]. Likewise, the single-cell RNA-seq technology BacDrop has shown that the intraspecies heterogeneity in *Klebsiella pneumoniae* is driven by MGEs [27].

To address the questions related to host–microbe co-evolution and the role of HGTs in human health, we need to bear in mind that it is a dynamic process. Studying HGT requires the development of technologies for real-time, high-precision, high-throughput recovery of microbial genomes, phages and plasmids to analyze their diverse characteristics. Moreover, multi-omics data, such as the metabolome from both the human genome and gut microbiome, can provide additional molecular information for understanding gene functionality and host–microbe interaction. We believe that the combination of large-scale metagenomics, omics and phenotype data will further help us understand the role of HGT in human health. Of course, experimental validation remains crucial to establish causal relationships and understand the underlying mechanisms.

In conclusion, technical advances will help us understand how HGT drives microbial adaptation, evolution and functional changes, and their relationship with the host's lifestyle and health. The potential to harness HGT to introduce and disseminate health-beneficial genes in the gut microbiome may also open up a new avenue for innovative disease prevention and treatment strategies.

## Supplementary Material

nwaf128_Supplemental_File

## References

[1] Smillie CS, Smith MB, Friedman J et al. Nature 2011; 480: 241–4. 10.1038/nature10571 22037308

[2] Zeevi D, Korem T, Godneva A et al. Nature 2019; 568: 43–8. 10.1038/s41586-019-1065-y 30918406

[3] Zahavi L, Lavon A, Reicher L et al. Nat Med 2023; 29: 2785–92. 10.1038/s41591-023-02599-8 37919437 PMC10999242

[4] Zhernakova DV, Wang D, Liu L et al. Nature 2024; 625: 813–21. 10.1038/s41586-023-06893-w 38172637 PMC10808065

[5] Brito IL . Nat Rev Microbiol 2021; 19: 442–53. 10.1038/s41579-021-00534-7 33846600

[6] Messerer M, Fischer W, Schubert S. PLoS One 2017; 12: e0179880. 10.1371/journal.pone.0179880 28732043 PMC5521745

[7] Wang H, Xia F, Xia Y et al. Bmc Genomics 2024; 25: 216. 10.1186/s12864-024-10146-z 38413855 PMC10898099

[8] Brito IL, Yilmaz S, Huang K et al. Nature 2016; 535: 435–9. 10.1038/nature18927 27409808 PMC4983458

[9] Groussin M, Poyet M, Sistiaga A et al. Cell 2021; 184: 2053–67. 10.1016/j.cell.2021.02.052 33794144

[10] Vatanen T, Jabbar KS, Ruohtula T et al. Cell 2022; 185: 4921–36. 10.1016/j.cell.2022.11.023 36563663 PMC9869402

[11] Dmitrijeva M, Tackmann J, Matias Rodrigues JF et al. Nat Ecol Evol 2024; 8: 986–98. 10.1038/s41559-024-02357-0 38443606 PMC11090817

[12] Song W, Wemheuer B, Zhang S et al. Microbiome 2019; 7: 1–14. 10.1186/s40168-0190649-y 30832740 PMC6399960

[13] Shoer S, Reicher L, Zhao C et al. Cell Host Microbe 2024; 32: 1744–57. 39353429 10.1016/j.chom.2024.08.017 PMC12060796

[14] Wang S, Jiang Y, Che L et al. Nucleic Acids Res 2024; 52: e61. 38884260 10.1093/nar/gkae515 PMC11317153

[15] Hsu TY, Nzabarushimana E, Wong D et al. Nat Microbiol 2025; 10: 94–111. 10.1038/s41564-024-01881-w 39747694

[16] Yu MK, Fogarty EC, Eren AM. Nat Microbiol 2024; 9: 830–47. 10.1038/s41564-024-01610-3 38443576 PMC10914615

[17] Fogarty EC, Schechter MS, Lolans K et al. Cell 2024; 187: 1206–22. 10.1016/j.cell.2024.01.039 38428395 PMC10973873

[18] Arnold BJ, Huang IT, Hanage WP. Nat Rev Microbiol 2022; 20: 206–18. 10.1038/s41579-021-00650-4 34773098

[19] Lee IPA, Eldakar OT, Gogarten JP et al. Trends Ecol Evol 2022; 37: 223–32. 34815098 10.1016/j.tree.2021.11.006

[20] Baas Becking LGM . Geobiologie; of Inleiding Tot De Milieukunde. The Hague, Netherlands: W. P. Van Stockum & Zoon, 1934.

[21] Valles-Colomer M, Blanco-Míguez A, Manghi P et al. Nature 2023; 614: 125–35. 10.1038/s41586-022-05620-1 36653448 PMC9892008

[22] Orakov A, Fullam A, Coelho LP et al. Genome Biol 2021; 22: 178. 10.1186/s13059-021-02393-0 34120611 PMC8201837

[23] Kuleshov V, Jiang C, Zhou W et al. Nat Biotechnol 2016; 34: 64–9. 10.1038/nbt.3416 26655498 PMC4884093

[24] Myeong NR, Choe Y-H, Shin SC et al. Sci Data 2024; 11: 1023. 39300163 10.1038/s41597-024-03875-z PMC11413225

[25] Grodner B, Shi H, Farchione O et al. Nat Microbiol 2024; 9: 2262–77. 10.1038/s41564-024-01735-5 38918467 PMC11371653

[26] Zheng W, Zhao S, Yin Y et al. Science 2022; 376: eabm1483. 10.1126/science.abm1483 35653470

[27] Ma P, Amemiya HM, He LL et al. Cell 2023; 186: 877–91. 10.1016/j.cell.2023.01.002 36708705 PMC10014032

