# Supplementary material for: Unveiling horizontal gene transfer in the gut microbiome: bioinformatic strategies and challenges in metagenomics analysis
Source: Natl Sci Rev. 2025 Apr 1;12(6):nwaf128. doi: 10.1093/nsr/nwaf128 (PMC12118462; doi:10.1093/nsr/nwaf128)
Supplement: nwaf128_Supplemental_File [file nwaf128_supplemental_file.docx]

***Supplementary Table 1. Tools for HGT detection.***

| **Strategy** | **Tools**(**Source)** | **Key Features** |
| --- | --- | --- |
| Sequence homology | PopCoGenT[1] (<https://github.com/philarevalo/PopCOGenT>)  Ranger-DTL[2] (<https://compbio.engr.uconn.edu/software/RANGER-DTL/>)  MetaCHIP[3] (<https://github.com/songweizhi/MetaCHIP>) | + Can be applied in closed communities with access to bacterial host information, which enables inference of transfer timing and direction and identification of individual genes or segments with high accuracy.  - Depends on quality and quantity of available genomic data.  - Cannot be effectively applied at the individual level without extensive culture and deep sequencing of each individual. |
| Split reads | Daisy[4] (<https://github.com/ktrappe/daisy>)  MGEfinder (https://github.com/bhattlab/MGEfinder)  LEMON[5] (<https://github.com/lichen2018/LEMON>)  LocalHGT[6] (https://github.com/deepomicslab/LocalHGT) | + Can identify new insertions, provide bacterial host information, determine direction of transfer, and detect complete transferred segments.  - Relies on selecting specific genomes.  - Faces challenges in accurately inferring timing of transfers.  - Overall accuracy is moderate. |
| Genomic Coverage | used in WAAFLE[7] (<https://github.com/biobakery/waafle>), Daisy (<https://github.com/ktrappe/daisy>), LEMON (<https://github.com/lichen2018/LEMON>), and LocalHGT (https://github.com/deepomicslab/LocalHGT) | - Not employed as the sole method for detecting HGT.  - Effectiveness varies based on presence of both donor and recipient in the sample.  + Provides a supplementary method to corroborate the occurrence of HGT. |
| Heterogenous regions | IslandViewer4[8-11] (https://www.pathogenomics.sfu.ca/islandviewer)  WAAFLE[7] (https://github.com/biobakery/waafle)  DICEP[12] (<https://github.com/Ronika19/DICEP>) | + Can detect HGT in individuals but represents only a fraction of the individual's total genetic makeup.  + Provides bacterial host information, partial determination of transfer direction, detection of one or more transferred genes with good accuracy, and identification of genes transferred by microorganisms absent from the broader microbial community.  - Cannot determine the timing of transfers.  - Scope of recoverable HGT information is limited. |
| Mobile genetic elements | geNomad[13] (https://github.com/apcamargo/genomad)  PlasX[14] (<https://github.com/michaelkyu/PlasX>) | + Detects gene transfer caused by specific mechanisms and identifies the transferred segments involved.  + Offers high accuracy.  + Knowing transfer mechanism can help elucidate results.  - Does not yield bacterial host information.  - Cannot determine the direction and timing of the transfer. |

**References:**

1. Arevalo P, VanInsberghe D, Elsherbini J, Gore J, Polz MF. A Reverse Ecology Approach Based on a Biological Definition of Microbial Populations. *Cell*. 2019/08/08/ 2019;178(4):820-834.e14. doi:<https://doi.org/10.1016/j.cell.2019.06.033>

2. Bansal MS, Kellis M, Kordi M, Kundu S. RANGER-DTL 2.0: rigorous reconstruction of gene-family evolution by duplication, transfer and loss. *Bioinformatics*. Sep 15 2018;34(18):3214-3216. doi:10.1093/bioinformatics/bty314

3. Song W, Wemheuer B, Zhang S, Steensen K, Thomas T. MetaCHIP: community-level horizontal gene transfer identification through the combination of best-match and phylogenetic approaches. *Microbiome*. 2019;7(1)doi:10.1186/s40168-019-0649-y

4. Trappe K, Marschall T, Renard BY. Detecting horizontal gene transfer by mapping sequencing reads across species boundaries. *Bioinformatics*. 2016;32(17):i595-i604.

5. Li C, Jiang Y, Li S. LEMON: a method to construct the local strains at horizontal gene transfer sites in gut metagenomics. *BMC bioinformatics*. 2019;20(Suppl 23):702.

6. Wang S, Jiang Y, Che L, Wang RH, Li SC. Enhancing insights into diseases through horizontal gene transfer event detection from gut microbiome. *Nucleic Acids Research*. 2024:gkae515.

7. Hsu TY, Nzabarushimana E, Wong D, et al. Profiling lateral gene transfer events in the human microbiome using WAAFLE. *Nature Microbiology*. 2025/01/02 2025;doi:10.1038/s41564-024-01881-w

8. Bertelli C, Laird MR, Williams KP, et al. IslandViewer 4: expanded prediction of genomic islands for larger-scale datasets. *Nucleic Acids Res*. Jul 3 2017;45(W1):W30-w35. doi:10.1093/nar/gkx343

9. Langille MGI, Hsiao WWL, Brinkman FSL. Evaluation of genomic island predictors using a comparative genomics approach. *BMC Bioinformatics*. 2008/08/05 2008;9(1):329. doi:10.1186/1471-2105-9-329

10. Hsiao W, Wan I, Jones SJ, Brinkman FSL. IslandPath: aiding detection of genomic islands in prokaryotes. *Bioinformatics*. 2003;19(3):418-420. doi:10.1093/bioinformatics/btg004

11. Waack S, Keller O, Asper R, et al. Score-based prediction of genomic islands in prokaryotic genomes using hidden Markov models. *BMC Bioinformatics*. 2006/03/16 2006;7(1):142. doi:10.1186/1471-2105-7-142

12. De R, Jani M, Azad RK. DICEP: An integrative approach to augmenting genomic island detection. *Journal of Biotechnology*. 2024/06/10/ 2024;388:49-58. doi:<https://doi.org/10.1016/j.jbiotec.2024.04.011>

13. Camargo AP, Roux S, Schulz F, et al. Identification of mobile genetic elements with geNomad. *Nature Biotechnology*. 2023/09/21 2023;doi:10.1038/s41587-023-01953-y

14. Yu MK, Fogarty EC, Eren AM. Diverse plasmid systems and their ecology across human gut metagenomes revealed by PlasX and MobMess. *Nature Microbiology*. 2024/03/01 2024;9(3):830-847. doi:10.1038/s41564-024-01610-3
